# Optimization and Prediction of Mechanical Properties of Additively Manufactured PLA/GNP Composites via Response Surface Methodology and Machine Learning Models

**DOI:** 10.3390/polym17212894

**Published:** 2025-10-29

**Authors:** Sundarasetty Harishbabu, Nashmi H. Alrasheedi, Borhen Louhichi, Santosh Kumar Sahu, Quanjin Ma

**Affiliations:** 1 School of Mechanical Engineering, VIT-AP University, Besides A.P. Secretariat, Amaravati 522237, Andhra Pradesh, India; 2 Department of Mechanical Engineering, College of Engineering, Imam Mohammad Ibn Saud Islamic University (IMSIU), Riyadh 11432, Saudi Arabia; 3 Engineering Sciences Research Center (ESRC), Deanship of Scientific Research, Imam Mohammad Ibn Saud Islamic University (IMSIU), Riyadh 11432, Saudi Arabia; 4 School of System Design and Intelligent Manufacturing, Southern University of Science and Technology, Shenzhen 518055, China

**Keywords:** 3D printing, process parameters, ANOVA, machine learning, SHAP analysis

## Abstract

This study investigates the optimization and prediction of mechanical properties in 3D-printed PLA composites reinforced with graphene nanoplatelets (GNP). The effects of GNP content (0, 2, and 5 wt.%), nozzle temperature (190–210 °C), print speed (20–60 mm/s), and layer thickness (0.15–0.35 mm) on tensile strength, Young’s modulus, and hardness were analyzed using a central composite design, at three print orientations (0°, 45°, and 90°). Compared to pure PLA, the incorporation of 5 wt.% GNP led to a 67% improvement in tensile strength, a 205% increase in Young’s modulus, and a 44% enhancement in hardness. Advanced machine learning models, such as XGBoost and Gaussian Process Regression, were employed for prediction, with R^2^ values exceeding 0.99 and MAPE below 4%. The models were validated using K-Fold Cross-Validation (K = 5), ensuring reliable and robust predictions while preventing overfitting. SHAP (Shapley Additive exPlanations) analysis indicated that GNP composition and layer thickness were the most influential factors, with SHAP values ranging between ±0.75. The Gaussian Process model outperformed both Linear Regression and XGBoost, achieving the highest R^2^ of 0.9900 ± 0.0021, the lowest MSE (0.6593 ± 0.1054), RMSE (0.812 ± 0.323), MAE (0.6755 ± 0.1123), MAPE (3.157% ± 0.320), and RRMSE (3.409% ± 0.513), highlighting its superior predictive accuracy and stability. This integrated methodology, combining experimental optimization, ANOVA, and interpretable machine learning, presents a promising and potentially robust strategy for optimizing the mechanical performance of GNP-reinforced PLA composites, emphasizing their potential for high-performance engineering applications.

## 1. Introduction

Three-dimensional (3D) printing, particularly Fused Deposition Modeling (FDM), has become a widely adopted manufacturing technique for producing polymer-based components with customized geometries and reduced material waste [1,2]. Among the various thermoplastics used in this process, polylactic acid (PLA) is one of the most popular choices due to its biodegradability, ease of processing, and compatibility with FDM systems. However, the mechanical limitations of pure PLA’s tensile strength are relatively low for demanding structural applications [3]. To overcome these drawbacks, PLA is often reinforced with nanomaterials to enhance its mechanical performance. The 3D printing process allows precise control over part design and manufacturing parameters, providing an effective platform for tailoring the properties of PLA-based composites [4]. As a result, these composites are being increasingly employed in biomedical devices, automotive components, packaging, and lightweight structural applications, highlighting their potential in advanced engineering fields [5,6]. Numerous studies have investigated PLA-based composites, focusing on processing techniques, reinforcement strategies, and property improvements. Reviewing these studies helps identify key trends and research gaps relevant to the present investigation. For instance, Jash et al. [7] found that reinforcing PLA with 2.5–5 wt.% ZnO nanoparticles and 10 wt.% plasticizer during 3D printing reduced the tensile strength from 46 MPa to 38 MPa at 5 wt.% ZnO. Additionally, elongation at break dropped from 7.2% to 4.3% at this ZnO concentration, which may be due to nanoparticle agglomeration or poor dispersion at higher concentrations. Dou et al. [8] demonstrated a remarkable enhancement in mechanical performance through continuous carbon fiber reinforcement, achieving tensile strength up to 243.5 MPa and stiffness of 25.8 GPa. The improvement was attributed to optimized printing parameters—low layer height (0.2 mm), narrow extrusion width (0.86 mm), high printing temperature (230 °C), and slow speed (50 mm/min)—which enhanced fiber–matrix bonding. Similarly, Arora et al. [9] found that incorporating betel nut shell-derived biocarbon significantly improved tensile and flexural strengths of PLA by 51.1% (at 0.1 wt.%) and 24.5% (at 0.025 wt.%), respectively, highlighting the potential of sustainable reinforcements. Further improvements were achieved through processing and surface modification. Kilinc et al. [10] observed that optimized printing parameters (layer height 0.6 mm, hatch spacing 0.4 mm) combined with atmospheric plasma treatment of aramid fibers enhanced fiber–matrix adhesion, increasing tensile and flexural strengths by ~7.8% and ~17.7%, respectively. Pandey et al. [11] reported that graphene oxide (GO) reinforcement enhanced tensile strength, modulus, and microhardness of PLA composites, with 0.5–1.25 wt.% GO yielding optimal mechanical performance. Similarly, Awwad et al. [12] found that adding graphene nanoplatelets (GNPs) to epoxy resin (up to 4.5 wt.%) improved stiffness and hardness by 7.6% but reduced tensile strength by 22% and fracture strain by 45.6%. Wear performance was significantly enhanced, with a 76% reduction in wear rate, a 37% decrease in friction coefficient, and a 22% lower interface temperature at 4.5 wt.%. Arunkumar et al. [13] developed PLA–Zn (80:20) composites with octet and cubic infill patterns, achieving a tensile strength of 13.9 MPa, a compressive strength of 60.58 MPa, an impact strength of 48.4 KJ/m^2^, and a hardness of 80 Shore D, making them suitable for lightweight load-bearing applications. Sutar et al. [14] studied the effects of graphene nanoplatelets (GNPs) with different thicknesses (H25: 15 nm and M25: 6–8 nm) on polypropylene (PP) nanocomposites. They found that adding up to 5 wt.% GNPs improved tensile strength, flexural strength, and impact strength by 30%, 25%, and 40%, respectively. M25 GNPs provided better dispersion and thermal stability than H25 GNPs, resulting in enhanced mechanical and thermal properties. The study concluded that thinner GNPs with higher surface area (M25) offer better reinforcement for PP nanocomposites. Peixoto et al. [15] developed PLA composites with Micrograf (PLA_M) and few-layer graphene (FLG), achieving a 14% exfoliation yield for FLG. For PLA_M composites, tensile and yield strengths remained relatively unchanged at 0.05 wt.% Micrograf, but the Young’s modulus decreased slightly. For PLA_FLG composites, at 0.05 wt.% FLG, tensile and yield strengths increased by around 7% and 6%, but at 0.5 wt.% FLG, mechanical properties declined due to agglomerates. Thermal stability decreased with FLG loading, and crystallinity increased slightly, suggesting potential for biomedical applications. Gharehbaghi et al. [16] investigated continuous glass fiber–reinforced PLA honeycombs, revealing superior residual strength and stiffness retention under fatigue loading. Tezel et al. [17] demonstrated that topology optimization of PLA structures achieved up to a 45% reduction in weight, while subsequent nickel coating enhanced the flexural strength by 60% (from 45 to 72 MPa) and Young’s modulus by 125%. Sztorch et al. [18] found that incorporating 0.25–2.5 wt.% SSQ-SH improved ductility, with elongation at break increasing by up to 56% and impact strength by 37%, though excessive loading led to particle agglomeration and reduced efficiency. Balasubramanian et al. [19] optimized 3D printing parameters for PLA/TCNP composites, reporting a maximum tensile strength of 45.67 MPa at 90% infill, 0° raster angle, and 3 mm/min strain rate. Seo et al. [20] studied the impact of graphene nanoplatelets (GNPs) on the dielectric properties and actuated strain of GNP-polydimethylsiloxane (PDMS) composites. They found that GNPs with larger particle sizes and thinner layers (M25) improved the dielectric constant to 4.26 at 1 kHz and achieved the highest actuated strain of 3.01% at <4 kV/mm. The M25 composites showed a 72% increase in Young’s modulus, making them promising materials for dielectric elastomer actuators. Finally, Khalifah Mahameru et al. [21] employed the Taguchi method to optimize PLA printing, identifying nozzle temperature and infill density as key factors, which resulted in an increase in tensile strength from 35 to 40 MPa.

Substantial progress has been made in reinforcing PLA with various fillers, including ZnO, carbon fibers, biocarbon, aramid fibers, TiO_2_, TCNP, and nickel coatings. Many studies have also explored the reinforcement of PLA with graphene nanoplatelets (GNPs). GNP is particularly attractive due to its two-dimensional structure, high aspect ratio, and exceptional mechanical properties. While mechanical performance improvements have been achieved by adjusting 3D printing parameters such as layer height, infill density, and printing speed, most previous works have been confined to experimental investigations, with few employing systematic optimization approaches like Response Surface Methodology (RSM) and ANOVA. The integration of machine learning for predictive analysis of PLA/GNP composites remains limited compared to experimental approaches, with many studies focusing primarily on traditional methods. The novelty of this study lies in its combination of experimental optimization (RSM and ANOVA) with interpretable machine learning (SHAP), offering a comprehensive approach to evaluate and predict the mechanical properties of PLA/GNP composites under varying printing conditions. Unlike existing studies, which primarily focus on mechanical performance or basic optimization, this study integrates systematic experimental optimization with machine learning to provide deeper interpretability, offering insights into how printing parameters influence composite properties. This innovative approach aims to develop a robust framework for designing high-performance, multifunctional PLA/GNP 3D-printed materials.

## 2. Materials and Methods

### 2.1. Materials

**Figure 1:**
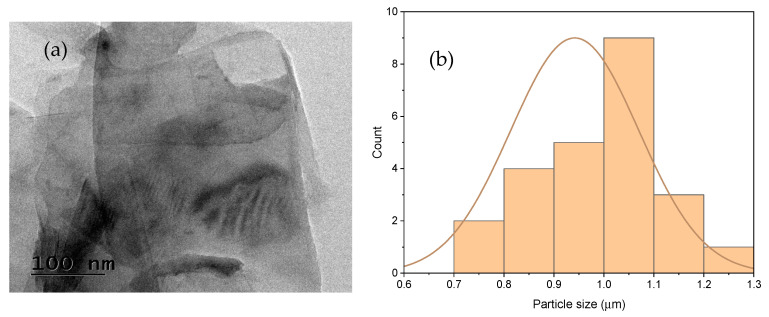
(**a**) TEM image of GNP; (**b**) Particle size distribution.

Polylactic acid (PLA) granules, with a density range of 1.20–1.30 g/cm^3^ and a melt flow index (MFI) of 10 g/10 min, were procured from Banka BioLoo Limited, Hyderabad, India. The reinforcement material, graphene nanoplatelets (GNP), was supplied by Adnano Technologies, Karnataka, India and is characterized by a platelet-like morphology, a thickness of approximately 5–10 nm, lateral dimensions of about 1 µm, a specific surface area of 110 m^2^/g, a bulk density of 0.166 g/cm^3^, and a purity of nearly 99%. The platelet-layered structure of the GNP was further confirmed through transmission electron microscopy (TEM), as illustrated in Figure 1a. Particle size analysis was performed and is shown in Figure 1b, indicating that the average particle size is close to 1 μm.

### 2.2. Experimental Design

The Response Surface Methodology (RSM) was employed to systematically design experiments aimed at optimizing the mechanical properties of PLA/GNP composites. This approach was selected for its capability to simultaneously evaluate the influence of multiple printing parameters, including filler composition (Table 1), as well as their interactions, while minimizing the number of experimental trials required. To achieve this, a Central Composite Design (CCD) with 22 randomized runs was implemented, enabling the development of quadratic models for accurate response prediction. This design not only captured both linear and non-linear effects of the parameters but also provided deeper insights into process–property relationships, offering a structured framework for performance optimization.

### 2.3. Fabrication of Samples

**Figure 2:**
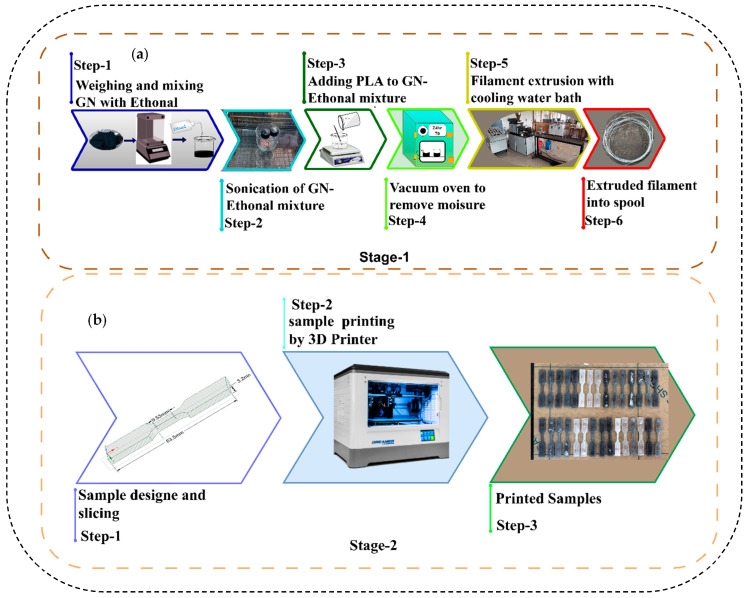
Fabrication of samples (**a**) filament extrusion; (**b**) sample printing.

The sample fabrication process consisted of two main stages, as illustrated in Figure 2. The first stage involved filament extrusion, with details summarized in Table 2 for both pure PLA and PLA/GNP composites, and illustrated in Figure 2a. Graphene nanoplatelets (GNP) were first weighed according to the required weight fraction using a precision scale (Shimadzu ATX-224, Shimadzu Co., Kyoto, Japan). The GNP nanofluid was prepared by dispersing 1 g of GNP in 20 mL of ethanol under magnetic stirring and mild heating to ensure uniform dispersion. PLA granules were gradually added to the dispersion while allowing complete ethanol evaporation, followed by drying in a vacuum oven (GR-58, Nano Tec, Chennai, India) at 70 °C for 24 h to remove residual moisture. Once dried, the PLA/GNP mixture was fed into an extruder, heated between 180 and 220 °C, and extruded through a die to form filaments with a nominal diameter of 1.75 mm. These filaments were cooled in a water bath, wound onto spools, and prepared for further processing. In the second stage, the extruded filaments were used to produce tensile and hardness specimens with a Flashforge Fused Deposition Modeling (FDM) 3D printer equipped with a 0.4 mm nozzle, providing precise control over layer height (minimum 0.12 mm), nozzle temperature (up to 250 °C), and print speed (up to 100 mm/s), ensuring consistent and accurate results. The tensile test specimens were fabricated according to ASTM D638 Type V [22], while the hardness test specimens followed ASTM E389 [23]. The geometries were designed in Ansys SpaceClaim 2019, as shown in Figure 2a,b, then converted into .STL format and imported into FlashPrint 5 software for slicing. “During slicing, parameters such as perimeter filler composition, nozzle temperature, print speed, and orientation angle in the XY plane were considered [24], and layer thickness (three levels) was varied, as detailed in Table 2, While a 100% infill density was targeted to ensure structural integrity, the infill density can only be approximate, and the print settings were adjusted to minimize these gaps and achieve the highest possible density for optimal strength. The final printing process was carried out on a Flashforge FDM printer, as illustrated in Figure 2b. Following fabrication, all specimens were carefully inspected for surface defects and measured for dimensional accuracy in compliance with ASTM standards before being subjected to mechanical testing.

### 2.4. Tensile Test

Tensile tests were conducted in accordance with ASTM D638 standards to evaluate the mechanical properties of the fabricated specimens. A Universal Testing Machine (UTM) (H10KL, Tinius Olsen India Pvt. Ltd., Noida, India) was employed to ensure precise control and accurate measurement during testing. The specimens were subjected to uniaxial tensile loading at a constant strain rate of 2 mm/min, a rate selected to promote uniform deformation and to enable reliable measurement of both tensile strength and Young’s modulus. Tensile strength represents the maximum stress a material can withstand before failure under stretching, whereas Young’s modulus reflects the material’s stiffness, indicating its resistance to elastic deformation under applied stress. The results obtained from these tests provide critical insights into the mechanical behavior of the composites under load, offering valuable information for their potential engineering applications. This testing approach is consistent with widely accepted practices in materials science research [25,26].

### 2.5. Hardness Test

Hardness testing of pure PLA and PLA/GNP composites was carried out using a Micro Vickers Hardness Tester (MC-AT, Fine Spavy Associates & Engineers Pvt. Ltd., Miraj, India) in accordance with ASTM standards. In this method, the Vickers Hardness Number (VHN) is determined based on the applied load (P, in kilograms-force, kgf) and the average diagonal length (W) of the indentation. A diamond pyramidal indenter with a face angle of 136° was used to produce micro-indentations on the specimen surfaces. Each specimen was subjected to an applied load of 0.05 kg for a dwell time of 10 s [27]. After unloading, the diagonals of the indentations were measured using an optical microscope, and the VHN was calculated using Equation (1) [28]. All measurements were conducted at room temperature, and the final hardness values were reported as the average of three indentations to ensure accuracy and consistency.

(1) $$\mathrm{VHN}=2\;\frac{\;\mathrm{Psin}\frac{136}{2}}{{dl}^{2}}$$

Here, VHN is the Vickers Hardness number, P is the applied load in kgf, and dl is the average length of the two diagonals.

### 2.6. Analysis of Variance

Analysis of Variance (ANOVA) is a widely used statistical technique that compares the means of multiple groups and determines the significance of various factors influencing a given response variable. In this study, ANOVA was performed using Design-Expert 13 software as part of the Response Surface Methodology (RSM) to assess the effects of independent factors on the response. RSM is a robust approach for process optimization, as it models and analyzes the relationships between multiple variables and their effects on outcomes. A key component of RSM, the Central Composite Design (CCD), was employed to investigate factor interactions, as well as linear and quadratic effects. Even without center points, the factorial structure of CCD enables the identification of main effects and quadratic terms. After fitting the experimental data to a second-order polynomial model, ANOVA was used to evaluate the statistical significance of each factor and interaction. This systematic approach allowed for the identification of the most influential parameters and the determination of their optimal levels, thereby improving overall process efficiency and performance [29].

### 2.7. Machine Learning

Machine learning (ML) offers a powerful data-driven approach to better understand and optimize the mechanical properties of 3D-printed PLA and PLA-GNP composites. Unlike traditional statistical techniques such as Analysis of Variance (ANOVA), ML can capture complex, non-linear relationships between processing parameters and material responses. In this study, several machine learning models were employed to predict key mechanical properties, including tensile strength, Young’s modulus, and impact strength, based on 3D printing parameters such as composition, nozzle temperature, printing speed, print angle, and layer thickness. To prevent overfitting, K-fold cross-validation (with K = 5) was used to evaluate the predictive accuracy of each model. This technique allowed for more reliable performance assessment by training and testing the model on different subsets of the data, which is particularly important given the small dataset (22 samples). Hyperparameter optimization was conducted for each model, with the Gaussian Process kernel selected based on the dataset’s characteristics and the XGBoost model optimized for learning rate and maximum tree depth. Additionally, the models were trained and tested on separate data splits to ensure proper generalization. The predictive performance of each model was evaluated using standard performance metrics. Mean Squared Error (MSE) and Root Mean Squared Error (RMSE) were used to quantify the average prediction error, while the Coefficient of Determination (R^2^) measured the proportion of variance explained by the model. To further assess predictive performance, additional error metrics such as Relative RMSE, Mean Absolute Error (MAE), and Mean Absolute Percentage Error (MAPE) were also calculated, as defined in Equations (2)–(7) [30,31]. The unusually high R^2^ values (above 0.99) observed in some models were carefully reviewed and validated to ensure the robustness of the results.

(2) $$R^{2}=1-\frac{\sum_{i=1}^{c}{{(f}_{i}-{\bar{f}}_{i})}^{2}}{\sum_{i=1}^{c}{{(f}_{i}-\bar{f})}^{2}}$$

(3) $$MSE=\frac{1}{c}\sum_{i=1}^{c}{{(f}_{i}-{\bar{f}}_{i})}^{2}$$

(4) $$RMSE=\sqrt{MSE}=\sqrt{({f_{i}-{\bar{f}}_{i})}^{2}}$$

(5) $$RRMSE=\frac{RMSE}{\bar{V}}=\frac{\sqrt{{{(f}_{i}-{\bar{f}}_{i})}^{2}}}{\bar{v}}\times 100$$

(6) $$MAE=\frac{1}{c}\sum_{i=1}^{c}\left|f_{i}-{\bar{f}}_{i}\right|$$

(7) $$MAPE=\frac{1}{c}\sum_{i=1}^{c}\left|\frac{f_{i}-{\bar{f}}_{i}}{f_{i}}\right|\times 100$$

In this context, c denotes the aggregate number of observations or trials. f_i_ represents the actual measured value for the cth trial, whereas $\bar{f}$ Reflects the mean of all actual values. Likewise, ${\bar{f}}_{i}$. Denotes the anticipated value associated with the ith observation.

#### 2.7.1. Linear Regression

Linear Regression (LR) is a statistical technique used to model the relationship between a dependent variable and one or more independent variables by representing the observed data with a linear equation. In simple linear regression, this relationship is expressed as shown in Equation (8). When multiple independent variables are involved, the method extends to multiple linear regression, as represented in Equation (9). This approach is widely used for predicting quantitative outcomes under the assumption of a linear relationship between predictors. The model is trained by minimizing the sum of squared errors between the predicted and actual values [32].

(8) $$R=+\varphi_{1}x+\delta \;(\mathrm{for}\mathrm{only}\mathrm{one}\mathrm{independent}\mathrm{variable})$$

(9) $$R=\varphi_{0}+\varphi_{1}x_{1}+\varphi_{1}x_{1}+------\varphi_{n}x_{n}+\delta \;(\mathrm{for multiple independent variables})$$

Here R is a dependent variable, x, x_1_, x_2_, ------ x_n_ are independent variables, $\varphi_{0}$ Is the intercept, $\varphi_{1}$, - - - -, $\varphi_{n}$ are the coefficients of the independent variables, δ Is the error term.

#### 2.7.2. Extreme Gradient Boosting (XG Boosting)

XGBoost is a robust machine learning algorithm that belongs to the gradient boosting family, building an ensemble of decision trees sequentially. Each successive tree is designed to correct the errors of its predecessors by combining its predictions with those of all previous trees, thereby improving accuracy and generalization while reducing the risk of overfitting. XGBoost achieves this by fitting a loss function to the data using gradient descent and incorporating a regularization term to penalize model complexity and further prevent overfitting. The overall objective function is defined in Equations (10) and (11). The algorithm’s strengths include its ability to efficiently process large datasets, handle missing data, and apply different loss functions for various tasks. XGBoost is highly regarded for its superior performance, efficiency, and scalability compared to most existing models for classification and regression problems. Additionally, its lightweight design and support for parallel computation result in faster training times compared to traditional gradient boosting methods [33].

(10) $$E\left(\emptyset \right)=\sum_{j=1}^{K}\left(l(R_{j}{{\bar{R}}_{j}}^{p}\right)+\sum_{n=1}^{p}\varphi \left(f_{n}\right)$$

(11) $$\varphi \left(f\right)=\partial T+\frac{1}{2}\delta {\left\|\in \right\|}^{2}$$

Here is a differentiable loss function, $f_{n}$ represents an individual regression tree at iteration n, T is the number of leaves in the tree, ∈ is the vector of scores on leaves, and ∂T and E(∅) are regularization parameters that penalize model complexity.

#### 2.7.3. Gaussian Process (GP)

A Gaussian Process (GP) is a non-parametric, probabilistic model used for regression and classification tasks, which defines a distribution over possible functions rather than assuming a predetermined functional form. This allows a GP to model the relationship between inputs and outputs with high flexibility, using a mean function m(x) to represent the expected value at each point and a covariance function K(X, X′) to describe the relationships between different points, as shown in Equation (12). For a given set of training data, the GP predicts outputs for new inputs by considering the joint distribution of the training and test points, which is assumed to follow a Gaussian distribution, as expressed in Equation (13). The predictive distribution of a GP is characterized by a mean, which provides the predicted value, and a variance, which quantifies the uncertainty associated with that prediction. This capability makes Gaussian Processes particularly powerful for modeling noisy and complex data where uncertainty quantification is critical. As a result, GPs are widely applied in areas such as time series forecasting, spatial data modeling, and optimization [34].

(12) $$f(x)\sim GP(m(x),k(x,x^{\prime }))$$

(13) $$\left[\begin{array}{c} R\\ \dot{R} \end{array}\right]\sim N\left(0,\left[\begin{array}{cc} K(X,X)+\propto_{N}^{2}I & K(X,\dot{X})\\ K(\dot{X},X) & K(\dot{X},\dot{X})+\propto_{N}^{2}I \end{array}\right]\right)$$

Here, f(x) is the function value at input x, m(x) is the mean function, and R is the vector of observed outputs for the training data. $\dot{R}$ is the vector of predicted outputs for the test data, and K(X, X) is the covariance matrix of the training inputs. $K(X,\dot{X})$ is the covariance matrix between training and test inputs $K(\dot{X},\dot{X})$ is the covariance matrix of the test inputs, $\propto_{N}^{2}$ Is the noise variance, and I is the identity matrix.

#### 2.7.4. SHapley Additive exPlanations (SHAP)

SHAP (Shapley Additive exPlanations) is a technique derived from cooperative game theory that quantifies the contribution of each feature to a model’s prediction. It does so by calculating the marginal contribution of a feature across all possible combinations of features, ensuring a fair and consistent allocation of contributions. Specifically, the SHAP value for a feature m is defined as the difference between the prediction with and without that feature, averaged over all possible subsets of features. In Linear Regression (LR), SHAP values correspond to the product of feature weights and their respective values. For XGBoost, they are computed by averaging the differences in predictions across decision trees when the feature is included versus excluded. In Gaussian Processes (GP), SHAP values represent the change in prediction caused by the inclusion or exclusion of a feature, thereby indicating its influence on the model’s posterior distribution. The general formula for SHAP values, which calculates the average marginal contribution of each feature across all feature subsets, is shown in Equation (14) [35].

(14) $$\partial_{j}(f)=\sum_{x\subseteq N\backslash \{m\}}\frac{\left|x\right|!(\left|N\right|-\left|x\right|-1)!}{\left|N\right|}[f(x\cup \{m\})-f(x)]$$

Here $\partial_{j}(f)$ Is the SHAP value for feature m, N is the set of all features, x is a subset of N excluding m, f(x) is the model’s prediction with the features in subset x, f(x∪{m}) is the model’s prediction with the features in x and feature m included.

## 3. Results and Discussion

### 3.1. Experimental Design

The optimization of the mechanical properties of PLA/GNP composites was performed using Response Surface Methodology (RSM) due to its efficiency in evaluating the effects of multiple printing parameters, including composition, as well as their interactions, while reducing the number of experimental trials required. The Central Composite Design (CCD) approach was adopted to develop quadratic models capable of accurately predicting responses, as detailed in Table 2. This design effectively captured both linear and non-linear effects of the parameters, offering valuable insights into process–property relationships and providing a systematic framework for optimizing composite performance.

**Table 2:** Experimental design.

Run	(F1) (Wt.%)	(F2) (°C)	(F_3_) (mm/s)	(F_4_) (Deg.)	(F_5_) (mm)	Tensile Strength (MPa)	Young’s Modulus (MPa)	Hardness (HV)
E1	Pure PLA	200	40	45	0.25	25.80	866.68	65.40
E2	5	190	20	90	0.35	34.80	1558.83	83.90
E3	Pure PLA	190	20	0	0.15	29.20	813.14	65.30
E4	2	200	40	0	0.25	24.60	655.11	65.80
E5	5	210	20	90	0.15	33.10	2064.15	82.20
E6	5	210	60	0	0.15	43.10	1418.12	77.90
E7	2	200	40	90	0.25	24.40	561.55	52.40
E8	5	210	20	0	0.35	37.90	2041.56	76.40
E9	2	200	40	45	0.25	18.80	408.96	62.60
E10	Pure PLA	210	60	90	0.15	28.50	1499.22	66.00
E11	2	200	40	45	0.35	14.10	509.71	75.10
E12	Pure PLA	210	60	0	0.35	17.20	759.56	80.60
E13	2	200	20	45	0.25	18.40	488.46	89.80
E14	Pure PLA	210	20	90	0.35	26.70	2647.26	90.25
E15	2	190	40	45	0.25	18.60	254.75	94.30
E16	2	210	40	45	0.25	15.50	737.27	82.70
E17	2	200	60	45	0.25	13.70	624.19	79.20
E18	2	200	40	45	0.15	13.00	1797.28	51.40
E19	5	200	40	45	0.25	21.60	1056.32	76.00
E20	5	190	60	90	0.15	21.40	767.72	70.20
E21	Pure PLA	190	60	90	0.35	15.40	458.54	62.90
E22	5	190	60	0	0.35	28.20	1546.76	77.00

### 3.2. Tensile Test

**Figure 3:**
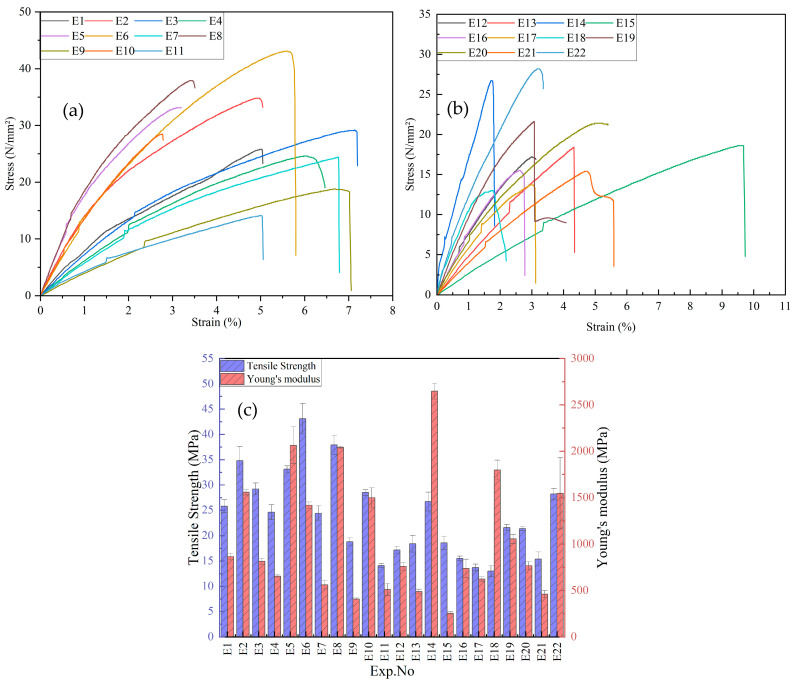
Experimental mechanical properties with L22 for PLA/GNP composites: (**a**) stress–strain curve(E1–E11), (**b**) stress–strain curve (E12–E22), and (**c**) tensile strength and Young’s modulus.

The tensile test results of PLA/GNP composites are presented in Figure 3a–c. Figure 3a,b illustrates the stress–strain behavior of the composites, revealing variations in stress response across the 22 experimental samples. The curves indicate that samples E7, E12, and E14 exhibit the highest tensile strength, suggesting superior load-bearing capacity, while samples E2, E9, and E19 show lower stress resistance and brittle failure—characterized by failure with minimal plastic deformation—at relatively low stress levels. Figure 3c presents the variation in tensile strength and Young’s modulus among the samples. The tensile strength ranges from 13.00 MPa to 43.10 MPa, with the highest values observed for E12 (43.10 MPa) and E5 (37.90 MPa), and the lowest for E19 (13.00 MPa) and E20 (14.10 MPa). Young’s modulus shows significant variation as well, with E14 (2647.26 MPa) and E5 (2064.15 MPa) demonstrating the highest stiffness, while E19 (254.75 MPa) and E20 (509.71 MPa) exhibit the lowest values. These variations highlight the strong influence of composition and processing parameters on the mechanical performance of PLA/GNP composites. Notably, compositions with 5% GNP and processing temperatures above 200 °C tend to exhibit the highest tensile strength, suggesting a potential relationship between these process parameters and the observed mechanical properties.

### 3.3. Hardness Test

**Figure 4:**
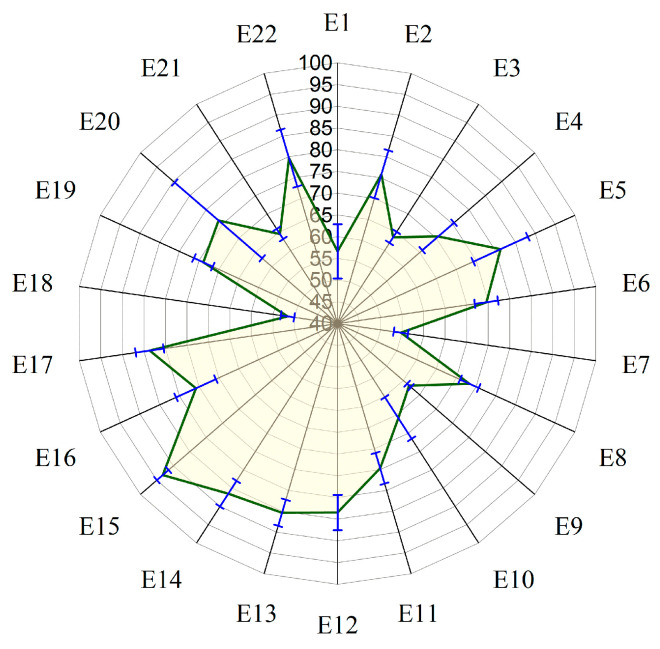
Hardness of PLA-GN composites with the L22 set of samples.

Figure 4 presents the hardness values of the PLA/GNP composites across the 22 samples, revealing notable variations in indentation resistance. The highest hardness was observed in sample E14 (94.30), followed closely by samples E13 (90.25) and E12 (89.80), indicating superior surface resistance and strength, while the lowest hardness values were found in samples E19 (51.40) and E7 (52.40), suggesting reduced surface resistance and lower mechanical performance. Hardness values ranged from 51.40 to 94.30, with a maximum variation of 42.90. These differences underscore the significant impact of composition and processing parameters on surface hardness. Specifically, higher hardness values are observed in composites with 5% GNP and higher processing temperatures (210 °C), such as E14 and E5, while lower hardness values are seen in composites with 2% GNP and lower processing temperatures (190 °C), like E19 and E7. This trend highlights the influence of GNP content and processing temperature on the mechanical properties of the PLA/GNP composites.

### 3.4. Analysis of Variance

#### 3.4.1. Tensile Strength

Table 3 presents the results of ANOVA analysis showing the effects of various printing parameters, including composition, on the tensile strength of PLA/GNP composites. The analysis reveals that composition (F1) is the most significant factor influencing tensile strength, with a *p*-value of 0.0342 and an F-value of 8.35, indicating its critical role in determining mechanical performance. Although temperature (F2), print speed (F3), and print angle (F4) do not individually exert significant effects on tensile strength (*p*-values of 0.7963, 0.3840, and 0.6226, respectively), their interactions with composition show notable significance. Specifically, the interactions between composition and temperature (F1 × F2) and composition and layer thickness (F1 × F5) are highly significant, with *p*-values of 0.0011 and 0.0070, respectively. Furthermore, non-linear effects from higher GNP loadings (e.g., 5 wt.%) reveal complex relationships between the factors and tensile strength. These non-linear mechanical responses can be attributed to agglomeration effects at higher GNP concentrations, where the poor dispersion of GNPs leads to a decrease in reinforcement efficiency, resulting in a plateau or decline in mechanical properties. The regression model explains 99.35% of the variation in tensile strength (R^2^ = 99.35%), demonstrating both an excellent fit and strong predictive capability.

**Figure 5:**
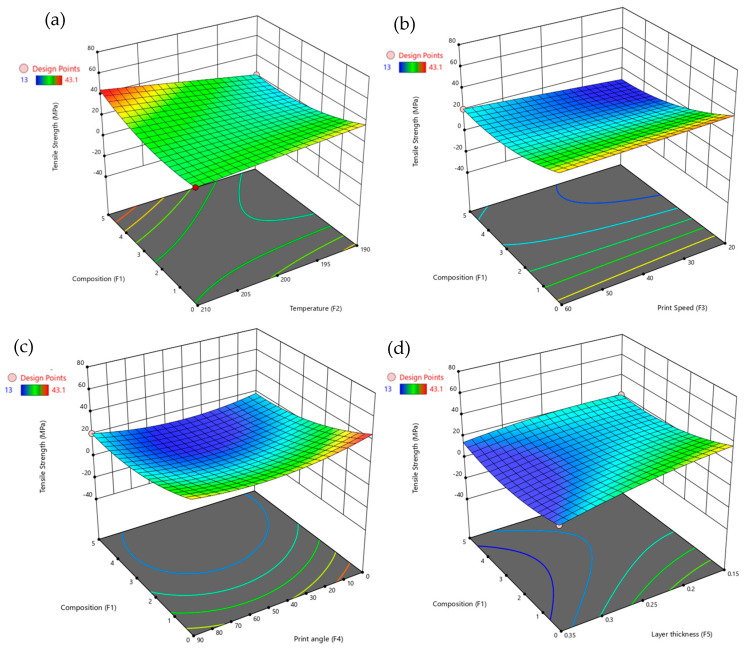
3D surface plots for tensile strength with composition vs. (**a**) temperature, (**b**) print speed, (**c**) print angle, and (**d**) layer thickness.

The 3D surface plots in Figure 5 illustrate the interactions between composition (F1) and other factors in influencing tensile strength. Figure 5a shows the relationship between composition and temperature (F2), revealing that tensile strength increases significantly for composition values above 0.5, particularly at higher levels. In contrast, increasing temperature slightly reduces tensile strength, especially at temperatures above 200 °C. Figure 5b, depicting composition versus print speed (F3), indicates that tensile strength is highest at lower print speeds (around 20 mm/s) and higher composition levels (0.5 and above). As print speed increases, particularly beyond 50 mm/s, tensile strength gradually decreases. Figure 5c, representing composition versus print angle (F4), shows a minor negative effect of print angle on tensile strength, especially at angles above 60°, where a decrease in tensile strength is observed. Nevertheless, composition continues to exert a positive influence, with higher composition levels consistently enhancing tensile strength even at larger print angles. Figure 5d, illustrating composition versus layer thickness (F5), demonstrates that tensile strength increases with both higher composition and greater layer thickness. The most notable strength improvement occurs when layer thickness approaches 0.15 mm, with the highest tensile strength recorded at composition levels around 0.5 and layer thickness between 0.12 and 0.15 mm. These plots underscore the dominant role of composition in determining tensile strength and highlight how its interactions with temperature, print speed, print angle, and layer thickness further influence the mechanical performance of PLA/GNP composites.

#### 3.4.2. Young’s Modulus

Table 4 presents the ANOVA results, illustrating the effects of printing parameters—composition (F1), temperature (F2), print speed (F3), print angle (F4), and layer thickness (F5)—on Young’s modulus of PLA/GNP composites. The analysis reveals that layer thickness (F5) is the most significant factor, with a *p*-value of 0.0002 and an F-value of 63.03, indicating a strong influence on stiffness. Temperature (F2) also shows a notable effect, with a *p*-value of 0.025 and an F-value of 8.82. Composition (F1), print speed (F3), and print angle (F4) do not individually exert significant influence on Young’s modulus, with *p*-values of 0.7963, 0.4185, and 0.4368, respectively. However, their interactions with composition and other factors—such as temperature, print speed, print angle, and layer thickness—are significant, demonstrating the importance of combined parameter effects. The model accounts for 99.02% of the variation in Young’s modulus (R^2^ = 99.02%), indicating an excellent fit, while the adjusted R^2^ of 96.57% confirms the model’s robustness after considering the number of predictors. These findings highlight the critical roles of temperature and layer thickness, both individually and in interaction with other factors, in determining the mechanical performance of PLA/GNP composites.

**Figure 6:**
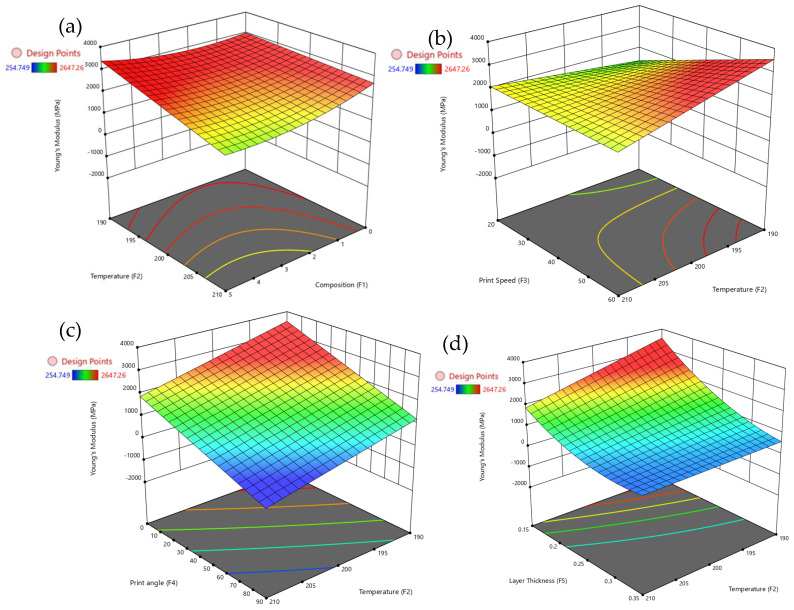
3D surface plots for Young’s modulus with temperature vs. (**a**) composition, (**b**) print speed, (**c**) print angle, and (**d**) layer thickness.

Figure 6 provides a comprehensive set of 3D surface plots illustrating how various factors influence Young’s modulus, with particular emphasis on temperature and layer thickness. Figure 6a shows that increasing composition above 0.5 enhances Young’s modulus, while higher temperatures (above 200 °C) tend to reduce stiffness. Figure 6b reveals that the highest Young’s modulus is achieved at lower print speeds (around 20 mm/s) combined with higher composition levels. However, increasing the print speed beyond 50 mm/s results in reduced stiffness, especially at elevated temperatures. Figure 6c demonstrates that print angles above 60° negatively affect Young’s modulus, although higher composition levels (above 0.5) can maintain or even improve stiffness regardless of the print angle. Figure 6d shows that increasing layer thickness, particularly in the range of 0.12 mm to 0.15 mm, significantly enhances stiffness. The highest Young’s modulus is observed at a composition of approximately 0.5 and a layer thickness between 0.12 mm and 0.15 mm. The composition plays a dominant role, temperature and layer thickness emerge as critical factors in optimizing the stiffness of PLA/GNP composites.

#### 3.4.3. Hardness Test

Table 5 presents the ANOVA results, highlighting how various printing parameters—such as composition (F1), temperature (F2), print speed (F3), print angle (F4), and layer thickness (F5)—affect hardness. The results show that layer thickness (F5) has the most significant effect on hardness, with a *p*-value of 0.0076 and an F-value of 11.74, indicating a strong influence. Composition (F1) also has a significant impact, with a *p*-value of 0.0204 and an F-value of 7.89. However, temperature (F2), print speed (F3), and print angle (F4) do not exhibit significant individual effects on hardness, with *p*-values of 0.9179, 0.1038, and 0.6555, respectively. Interestingly, interactions between composition and layer thickness (F1*F5), as well as squared terms for temperature (F2^2^), print speed (F3^2^), print angle (F4^2^), and layer thickness (F5^2^), significantly influence hardness, indicating that their combined effects and non-linear relationships are critical in determining hardness. The model explains 86.20% of the variation in hardness, indicating a strong fit, but the adjusted R^2^ of 67.80% suggests that the model might be prone to overfitting, particularly considering the small sample size. This discrepancy between R^2^ and adjusted R^2^ is noted to contextualize the model’s reliability. These findings underscore the significant role of composition and layer thickness in determining hardness, both individually and through their interactions, highlighting their importance in optimizing the mechanical properties of PLA/GNP composites.

**Figure 7:**
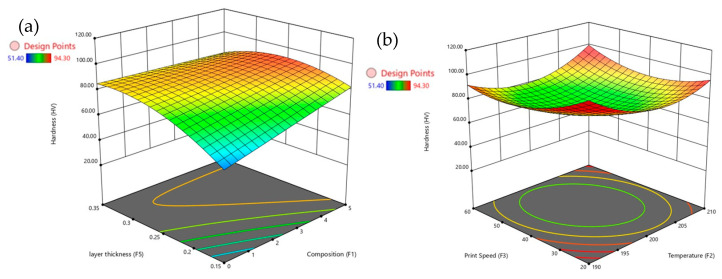

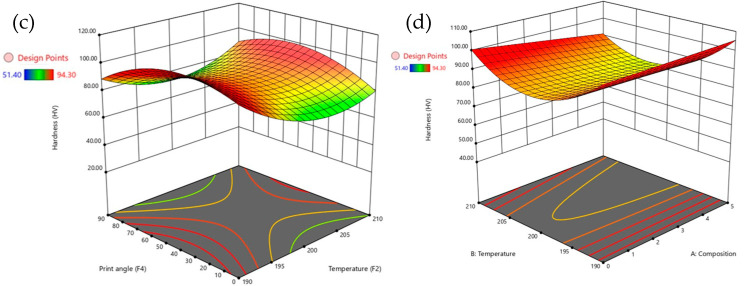
3D surface plots for hardness with composition vs. (**a**) temperature, (**b**) print speed, (**c**) print angle, and (**d**) layer thickness.

Figure 7 provides a detailed set of 3D surface plots illustrating how various factors influence hardness, with a particular emphasis on composition. Figure 7a shows that increasing composition significantly enhances hardness, especially when the layer thickness is between 0.15 mm and 0.20 mm. However, larger layer thicknesses tend to reduce hardness even at higher composition levels. Figure 7b reveals that moderate print speeds, around 30–40 mm/s, combined with higher temperatures above 200 °C, result in the highest hardness values. In contrast, very low temperatures or excessively high print speeds lead to a reduction in hardness. Figure 7c demonstrates that print angles above 60° negatively affect hardness, although higher compositions can partially offset this decrease. Figure 7d shows that hardness is maximized at compositions above 0.5 and moderate extrusion temperatures between 200 °C and 205 °C, while excessively high temperatures tend to diminish hardness. The composition emerges as the dominant factor affecting hardness, but its influence is strongly modulated by layer thickness and temperature, with print speed and print angle playing secondary roles in determining the surface hardness of PLA/GNP composites.

**Figure 8:**
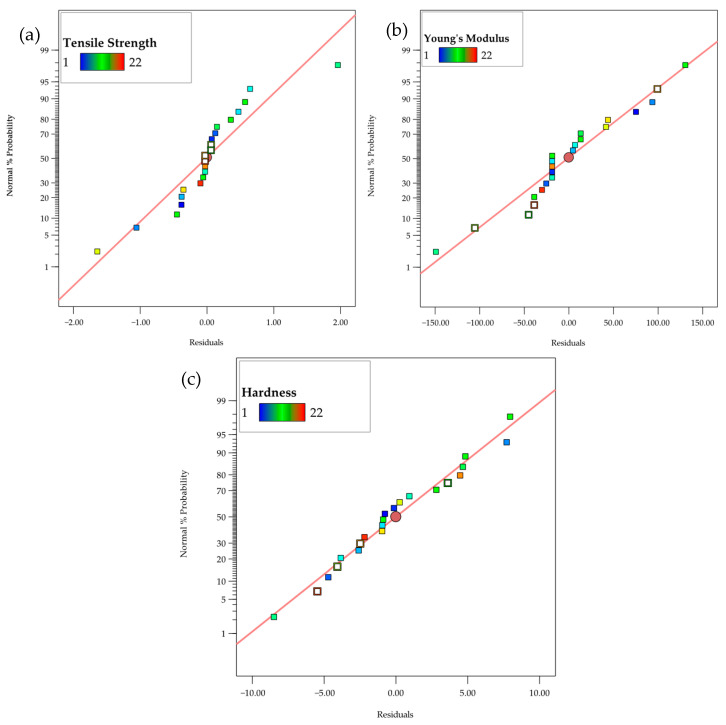
Normal residual plots for (**a**) Tensile strength, (**b**) Young’s modulus, (**c**) Hardness.

Figure 8a–c shows the residual plots for three material properties: tensile strength, Young’s modulus, and hardness. The *x*-axis shows the residuals, which are the differences between observed and predicted values, while the *y*-axis displays the normalized percentage probability, indicating the distribution of these residuals. The red line represents the expected linear relationship, with data points color-coded to show a continuous variable. For tensile strength, the residuals range from about −2.0 to 2.0, with a percentage probability from approximately 5% to 50%. For Young’s modulus, the residuals range from −15 to 15, with a probability between 10% and 40%. For hardness, the residuals range from −5 to 5, with a probability distribution from 10% to 60%. All three properties show residuals scattered randomly around the red line, indicating a well-calibrated regression model. There are no clear patterns or outliers, suggesting the model accurately captures the relationships between the material properties and their residuals. The lack of trends or clusters further supports assumptions of linearity and homoscedasticity, confirming the model fits the data well with minimal differences between predicted and actual values.

### 3.5. Machine Learning

#### 3.5.1. Tensile Strength

**Figure 9:**
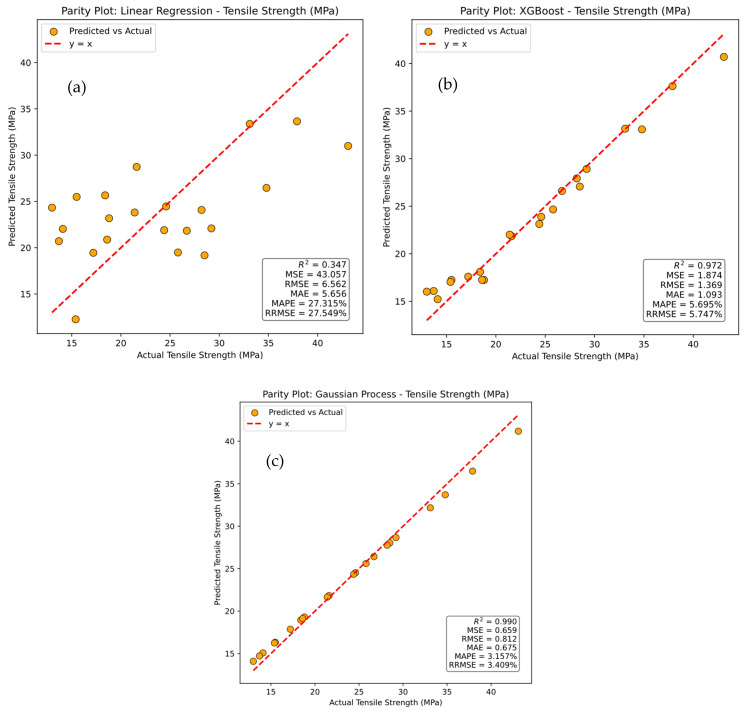
Parity plots for actual versus prediction for tensile strength of (**a**) linear regression, (**b**) XG Boost, and (**c**) Gaussian process.

The parity plots shown in Figure 9a–c, comparing actual versus predicted tensile strength for the Linear Regression, XGBoost, and Gaussian Process models, reveal clear differences in model performance. In each plot, the red dotted line represents y = x, indicating perfect agreement between predicted and actual values, while the dots represent individual sample predictions. Significant scatter around this line indicates discrepancies between predicted and actual results. Figure 9a shows that the Linear Regression model exhibits considerable prediction error, as reflected in its performance metrics: a coefficient of determination (R^2^) of 34.6%, Mean Squared Error (MSE) of 43.056, Root Mean Squared Error (RMSE) of 6.56, Mean Absolute Error (MAE) of 5.656, Mean Absolute Percentage Error (MAPE) of 27.314%, and Relative Root Mean Squared Error (RRMSE) of 27.549%. These results highlight its limited capability to capture the variance in the data. In Figure 9b, the XGBoost model demonstrates a significantly stronger correlation between predicted and actual values, with R^2^ = 97.16%, MSE = 1.874, RMSE = 1.37, MAE = 1.093, MAPE = 5.70%, and RRMSE = 5.75%, indicating a substantial improvement over Linear Regression. Figure 9c illustrates that the Gaussian Process model achieves the highest accuracy, with predicted values closely matching actual values. This model records the best performance, with R^2^ = 99.00%, MSE = 0.66, RMSE = 0.81, MAE = 0.68, and MAPE = 3.16%, confirming its superior predictive capability. It is observed that the Gaussian Process model outperforms both Linear Regression and XGBoost, delivering the most accurate predictions with the lowest error metrics for tensile strength.

**Figure 10:**
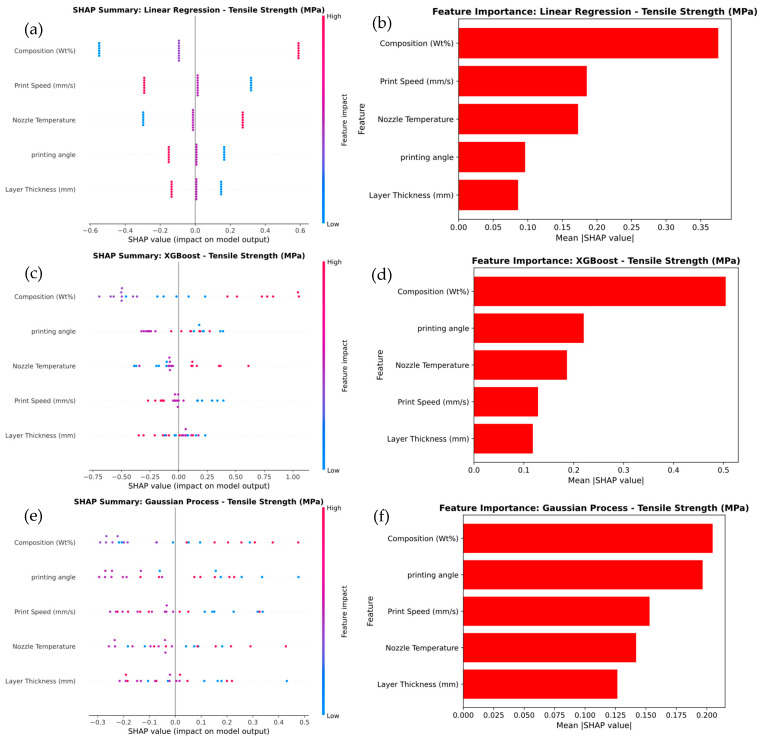
SHAP Analysis of summary and Feature Importance for tensile strength prediction of (**a**,**b**) linear regression, (**c**,**d**) XG Boost, and (**e**,**f**) Gaussian process.

The SHAP analysis presented in Figure 10 illustrates the feature importance for tensile strength prediction across different models, including Linear Regression, XGBoost, and Gaussian Process. In the Linear Regression model in Figure 10a, Composition (wt.%) emerges as the most influential feature, with SHAP values ranging from −0.64 to +0.64, indicating a strong impact on the prediction. The corresponding feature importance plot in Figure 10b confirms this, assigning Composition (wt.%) the highest importance score of 0.5. Other features, such as Print Speed (0.2) and Nozzle Temperature (0.1), have comparatively lower contributions. For the XGBoost model in Figure 10c, Composition (wt.%) again dominates but shows a broader range of SHAP values from −0.75 to +0.75, reflecting greater variability and influence. Notably, Layer Thickness emerges as a more significant feature with an importance score of 0.35, as shown in Figure 10d, highlighting XGBoost’s ability to capture complex, non-linear relationships that Linear Regression cannot. Similarly, in the Gaussian Process model in Figure 10e, Composition (wt.%) remains the most dominant feature, while Layer Thickness gains even more relevance, with an importance score of 0.3 in Figure 10f and SHAP values ranging from −0.5 to +0.5. This trend demonstrates that as model complexity increases—from Linear Regression to XGBoost and Gaussian Process—the importance of Layer Thickness grows, reflecting these models’ enhanced ability to capture intricate, non-linear interactions. It was noted that, the Composition (wt.%) consistently plays a central role across all models, more advanced models such as XGBoost and Gaussian Process emphasize the increased significance of Layer Thickness, underlining their superior capability in modeling complex relationships in tensile strength prediction.

**Figure 11:**
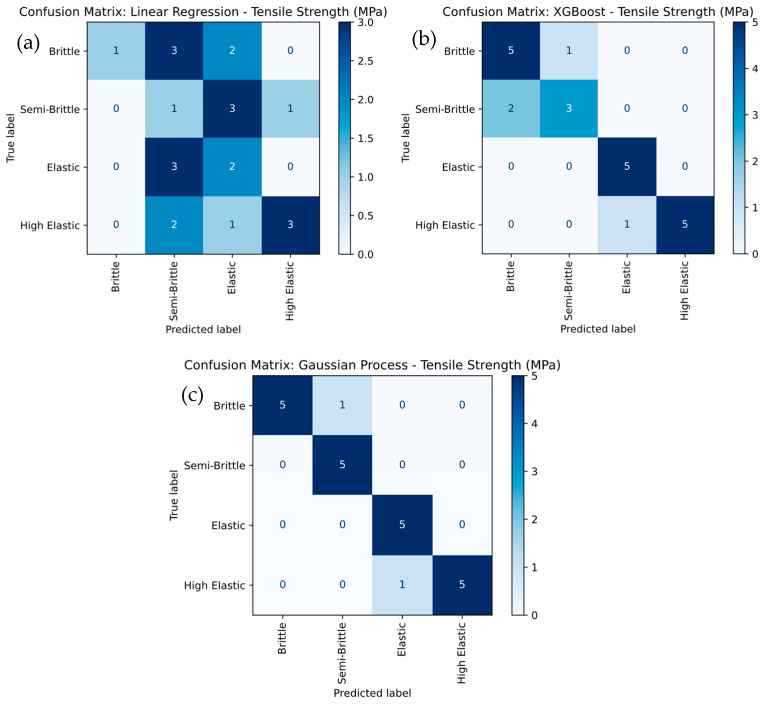
Confusion-Matrix Comparison for Tensile Strength Severity Levels for (**a**) linear regression, (**b**) XG Boost, and (**c**) Gaussian Process.

Figure 11 provides a detailed comparison of the tensile strength classification of PLA-GN composites using different machine learning models, including Linear Regression, XGBoost, and Gaussian Process. In Figure 11a, the Linear Regression model exhibits the weakest performance, with an overall accuracy of 56.25%. It struggles to accurately classify the Brittle, Semi-Brittle, and Elastic categories, with frequent misclassifications—particularly between Brittle and Semi-Brittle classes. The low recall values for Brittle (25%) and Elastic (40%) further highlight these limitations. In contrast, the XGBoost model shown in Figure 11b demonstrates strong performance, achieving 100% overall accuracy. It correctly predicts Elastic and High Elastic categories with perfect recall, though it misclassifies Semi-Brittle as Brittle in two instances. Specifically, XGBoost achieves 100% recall for Elastic and 83.3% for High Elastic, while Semi-Brittle attains a recall of 60%, showing significant improvement over Linear Regression. Figure 11c shows that the Gaussian Process model outperforms both Linear Regression and XGBoost, achieving 100% overall accuracy.

It attains perfect recall for Elastic and Semi-Brittle categories, and 83.3% recall for Brittle and High Elastic. This superior performance demonstrates the Gaussian Process model’s exceptional ability to handle complex, non-linear relationships, resulting in higher classification accuracy and precision. It was observed that the Gaussian Process model emerges as the best-performing approach, followed by XGBoost, while Linear Regression is the least effective due to its inability to capture non-linear decision boundaries effectively.

#### 3.5.2. Young’s Modulus

**Figure 12:**
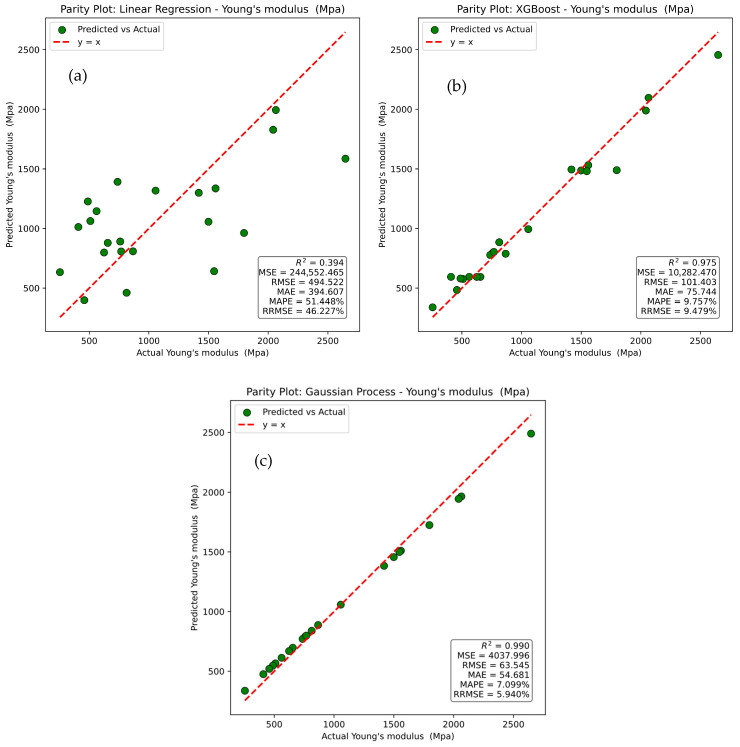
Parity plots for actual versus prediction for Young’s modulus of (**a**) linear regression, (**b**) XG Boost, and (**c**) Gaussian process.

The parity plots in Figure 12 compare the actual values of Young’s modulus with those predicted by different machine learning models, including Linear Regression, XGBoost, and Gaussian Process. The red dashed line represents the ideal relationship y = x, while the green dots correspond to individual data points, with deviations from the line indicating prediction errors.

In Figure 12a, the Linear Regression model exhibits a wide scatter of points around the reference line, reflecting poor predictive capability. This is confirmed by an R^2^ value of 0.394, a Mean Squared Error (MSE) of 244,552.465, a Root Mean Squared Error (RMSE) of 494.522, a Mean Absolute Error (MAE) of 394.607, a Mean Absolute Percentage Error (MAPE) of 51.448%, and a Relative Root Mean Squared Error (RRMSE) of 46.227%, all indicating substantial prediction errors. In Figure 12b, the XGBoost model shows a much closer alignment of predicted values with actual values, demonstrating strong predictive performance. It achieves an R^2^ of 0.975, an MSE of 10,282.470, an RMSE of 101.403, an MAE of 75.744, a MAPE of 9.757%, and an RRMSE of 9.479%, highlighting its robust ability to capture non-linear relationships. Figure 12c shows that the Gaussian Process model delivers the highest predictive accuracy, with predicted points clustering most closely along the reference line. It attains an R^2^ of 0.990, an MSE of 4037.996, an RMSE of 63.545, an MAE of 54.681, an MAPE of 7.099%, and an RRMSE of 5.940%, confirming its superiority over both Linear Regression and XGBoost in predicting Young’s modulus. It was noted that Gaussian Process model demonstrates the best predictive capability, followed by XGBoost, while Linear Regression performs least effectively due to its limited capacity to model non-linear dependencies.

**Figure 13:**
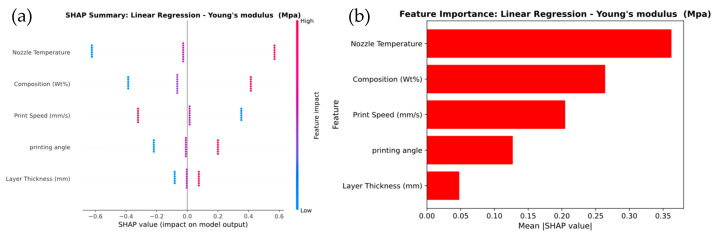

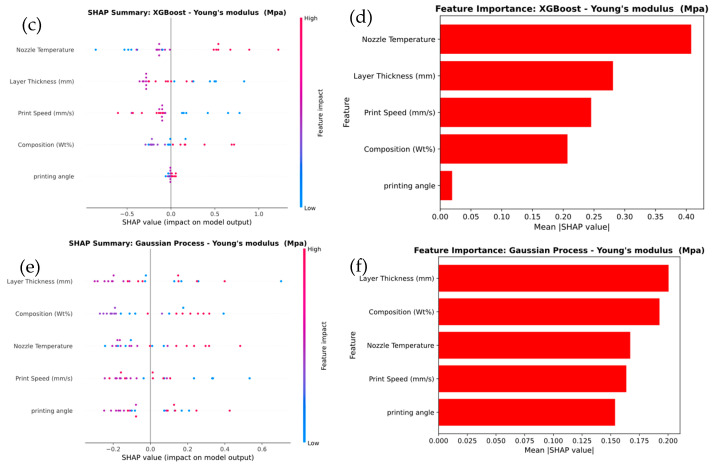
SHAP Analysis of Feature Importance for Young’s modulus prediction of (**a**,**b**) linear regression, (**c**,**d**) XG Boost, and (**e**,**f**) Gaussian process.

Figure 13 illustrates the feature importance for predicting Young’s modulus across the Linear Regression, XGBoost, and Gaussian Process models. In Figure 13a, the Linear Regression model shows that Nozzle Temperature and Composition (wt.%) are the most influential features, with SHAP values ranging from −0.6 to +0.6. This is further confirmed by the feature importance plot in Figure 13b, which assigns the highest importance scores to these features. In Figure 13c, the XGBoost model highlights a stronger influence of Nozzle Temperature and Layer Thickness, with SHAP values ranging from −1.0 to +1.0. The feature importance plot in Figure 13d confirms this, demonstrating that XGBoost captures more variation and non-linear interactions compared to Linear Regression.

For the Gaussian Process model, shown in Figure 13e, Layer Thickness emerges as the most influential feature, with SHAP values ranging from −0.2 to +0.6. Nozzle Temperature and Composition (wt.%) also have notable impacts. This observation is supported by the feature importance plot in Figure 13f, where Layer Thickness receives the highest importance score, followed by Composition (wt.%) and Nozzle Temperature. It was observed that the SHAP analysis demonstrates that Layer Thickness and Nozzle Temperature are critical factors in predicting Young’s modulus across all models. XGBoost captures more complex feature relationships, while the Gaussian Process model emphasizes the dominant role of Layer Thickness in determining stiffness.

**Figure 14:**
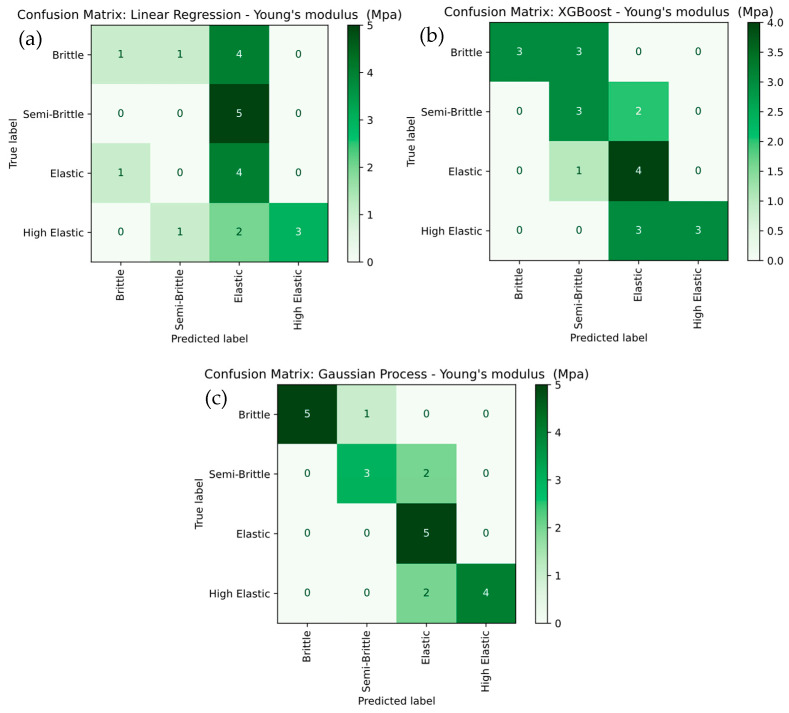
Confusion-Matrix Comparison for Young’s modulus Severity Levels for (**a**) linear regression, (**b**) XG Boost, and (**c**) Gaussian Process.

Figure 14 presents a detailed comparison of Young’s modulus severity level classification using Linear Regression, XGBoost, and Gaussian Process models. In Figure 14a, the Linear Regression model exhibits the weakest performance, with frequent misclassifications among the Brittle, Semi-Brittle, and Elastic categories. It particularly misclassifies Brittle as Elastic and Semi-Brittle as Brittle, resulting in low overall accuracy. The low recall values for Brittle (25%) and Elastic (40%) further highlight the model’s limitations in capturing the underlying data patterns. In contrast, the XGBoost model shown in Figure 14b delivers noticeably better performance, achieving higher accuracy, especially for the Elastic and High Elastic categories. However, it still misclassifies Semi-Brittle as Brittle in some cases. XGBoost achieves perfect recall for Elastic (100%) and strong recall for High Elastic (83.3%), while Semi-Brittle achieves a recall of 60%, indicating a clear improvement over Linear Regression. The Gaussian Process model in Figure 14c outperforms both Linear Regression and XGBoost, achieving perfect classification for Elastic and Semi-Brittle categories and 83.3% recall for both Brittle and High Elastic. Its superior ability to model complex, non-linear relationships results in the highest overall accuracy and precision, with perfect classification for the most critical categories. The results noted that Gaussian Process model demonstrates the best classification performance, followed by XGBoost, while Linear Regression performs the least effectively due to its inability to capture non-linear decision boundaries in the data.

#### 3.5.3. Hardness Test

**Figure 15:**
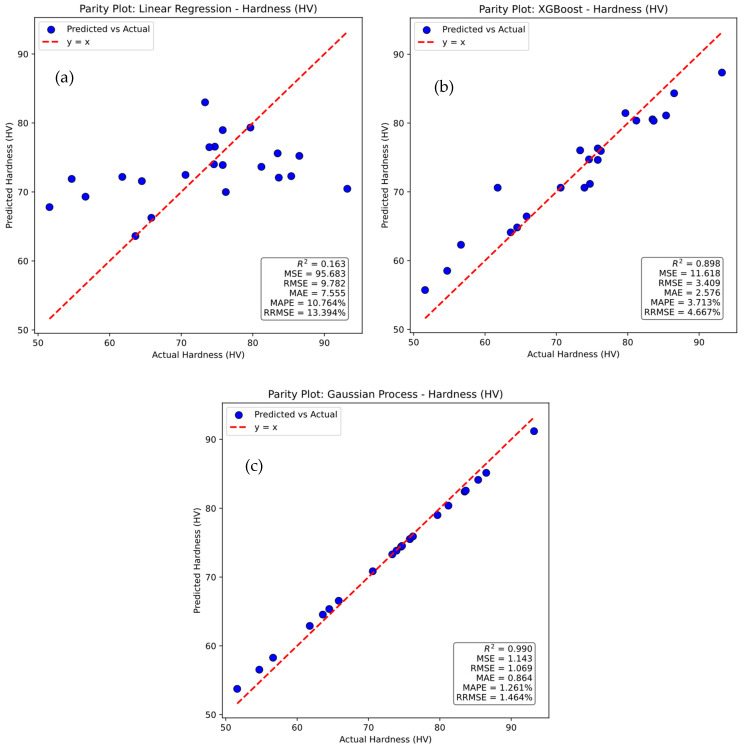
Parity plots for actual versus prediction for Hardness of (**a**) linear regression, (**b**) XG Boost, and (**c**) Gaussian process.

The parity plots in Figure 15 compare the actual values of hardness with those predicted by machine learning approaches using linear regression, XGBoost, and Gaussian Process models. The red dashed line represents the ideal y = x relationship, and the blue dots denote individual data points, where deviations from the line indicate prediction errors. In Figure 15a, the linear regression model shows large scatter around the reference line, reflecting poor predictive capability with an R^2^ of 0.163, a Mean Squared Error of 95.683, a Root Mean Squared Error of 9.782, a Mean Absolute Error of 7.555, a Mean Absolute Percentage Error of 10.764 percent, and a Relative Root Mean Squared Error of 13.394 percent. In Figure 15b, the XGBoost model demonstrates much stronger predictive performance, with closer alignment between predicted and actual values, achieving an R^2^ of 0.898, a Mean Squared Error of 11.618, a Root Mean Squared Error of 3.409, a Mean Absolute Error of 2.576, a Mean Absolute Percentage Error of 3.713 percent, and a Relative Root Mean Squared Error of 4.667 percent. In Figure 15c, the Gaussian Process model provides the best results, with points tightly clustered along the reference line and the highest predictive accuracy, reflected by an R^2^ of 0.990, a Mean Squared Error of 1.143, a Root Mean Squared Error of 1.069, a Mean Absolute Error of 0.864, a Mean Absolute Percentage Error of 1.261 percent, and a Relative Root Mean Squared Error of 1.464 percent. These results confirm the superiority of the Gaussian Process model over linear regression and XGBoost in predicting hardness.

**Figure 16:**
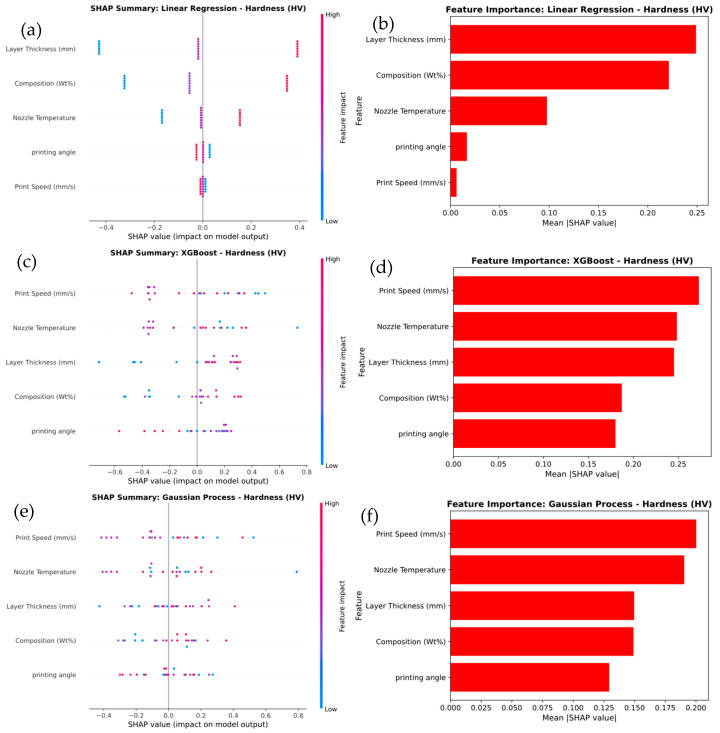
SHAP Analysis of Feature Importance for Hardness prediction of (**a**,**b**) linear regression, (**c**,**d**) XG Boost, and (**e**,**f**) Gaussian process.

Figure 16 presents a SHAP analysis of feature importance for hardness prediction across three machine learning models: Linear Regression, XGBoost, and Gaussian Process. In the Linear Regression model in Figure 16a, Layer Thickness and Composition (wt.%) emerge as the most influential features, with SHAP values indicating their strong impact on predictions. This is confirmed by the feature importance plot in Figure 16b, which shows Layer Thickness as the dominant feature, followed by Composition (wt.%). Other features, such as Print Speed and Nozzle Temperature, contribute comparatively less to the model’s output. In the XGBoost model in Figure 16c, Layer Thickness remains the most significant feature, but Print Speed also emerges as a major contributor, exhibiting a broader range of SHAP values that highlight greater variability in its influence. The feature importance plot in Figure 16d confirms that both Layer Thickness and Print Speed have the highest contributions to the predictions, while the other factors show relatively lower importance. For the Gaussian Process model in Figure 16e, Layer Thickness continues to be the most impactful feature; however, the influence of Print Speed becomes even more pronounced. This is reflected in the SHAP value distribution and the feature importance plot in Figure 16f, where Print Speed and Layer Thickness exhibit the greatest effect, while other features play a lesser role.

The results show that the Layer Thickness consistently plays a critical role across all models, but more complex models, such as XGBoost and Gaussian Process, underscore the increased significance of Print Speed. This reflects their enhanced ability to capture intricate, non-linear relationships that simpler models like Linear Regression cannot fully capture.

**Figure 17:**
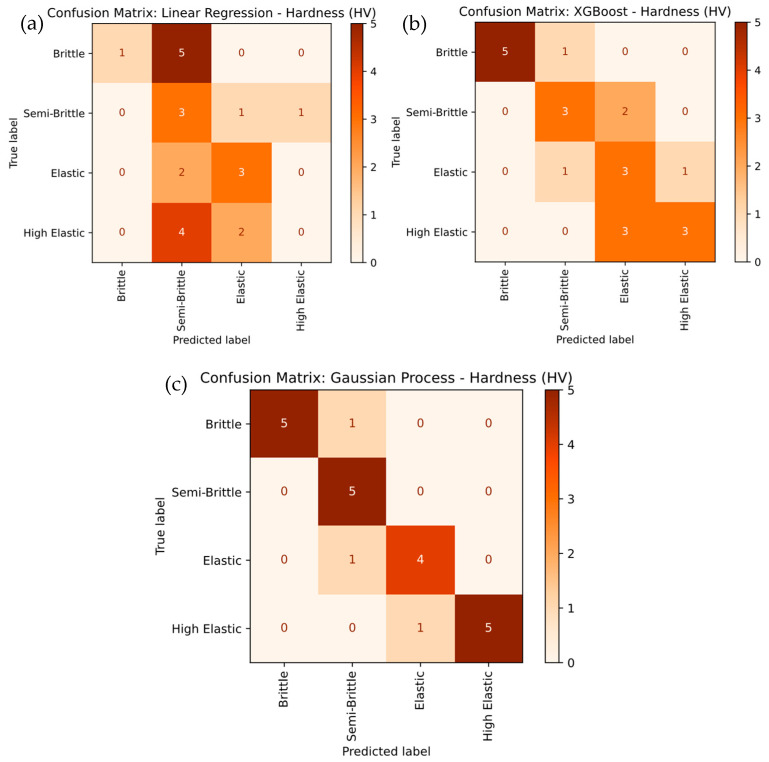
Confusion-Matrix Comparison for Hardness Severity Levels for (**a**) linear regression, (**b**) XG Boost, and (**c**) Gaussian Process.

Figure 17 presents a detailed comparison of hardness severity classification using Linear Regression, XGBoost, and Gaussian Process models. The Linear Regression model, shown in Figure 17a, demonstrated the weakest performance, with substantial misclassifications, particularly in the Semi-Brittle and High Elastic categories. Specifically, the model misclassified Semi-Brittle as Brittle in three cases and Elastic as Semi-Brittle in two instances, leading to lower overall accuracy. The recall values further highlight these limitations, with Brittle at 20% and Semi-Brittle at 60%. In contrast, the XGBoost model in Figure 17b showed improved performance, achieving higher accuracy, especially for the Elastic and High Elastic categories, although some misclassifications of Semi-Brittle as Brittle still occurred. XGBoost achieved strong recall for Elastic (75%) and High Elastic (83.3%), while Semi-Brittle maintained a recall of 60%. The Gaussian Process model, presented in Figure 17c, outperformed both alternatives, achieving perfect classification for the Semi-Brittle and Elastic categories. It also achieved a recall of 83.3% for both the Brittle and High Elastic categories. This superior performance is attributed to its ability to capture complex, non-linear relationships, resulting in the highest overall prediction accuracy with 100% correct classification for key categories. It was noted that the Gaussian Process model proved to be the most effective, followed by XGBoost, while Linear Regression showed the poorest performance due to its inability to capture non-linear decision boundaries effectively.

#### 3.5.4. K-Fold Cross-Validation and Correlation

The performance of Linear Regression, XGBoost, and Gaussian Process Regression models was evaluated using K-Fold Cross-Validation (K = 5). The Linear Regression model showed relatively poor performance, with an R^2^ of 0.3469 ± 0.0231, indicating limited variance explained, and high prediction errors, as reflected by the MSE (43.057 ± 3.257), RMSE (6.562 ± 0.788), MAE (5.656 ± 0.923), and MAPE (27.315% ± 2.457). In contrast, the XGBoost model demonstrated significantly better performance, with an R^2^ of 0.9716 ± 0.0103, MSE (1.874 ± 0.342), RMSE (1.369 ± 0.299), MAE (1.093 ± 0.158), MAPE (5.695% ± 0.476), and RRMSE (5.747% ± 0.428), indicating more accurate predictions and lower error rates. However, the Gaussian Process model outperformed both, achieving the highest R^2^ of 0.9900 ± 0.0021, with the lowest MSE (0.6593 ± 0.1054), RMSE (0.812 ± 0.323), MAE (0.6755 ± 0.1123), MAPE (3.157% ± 0.320), and RRMSE (3.409% ± 0.513), highlighting its superior predictive accuracy and stability. The low standard deviations for the Gaussian Process across all metrics indicate its robustness, making it the most reliable model for predicting the mechanical properties of 3D-printed composites.

**Figure 18:**
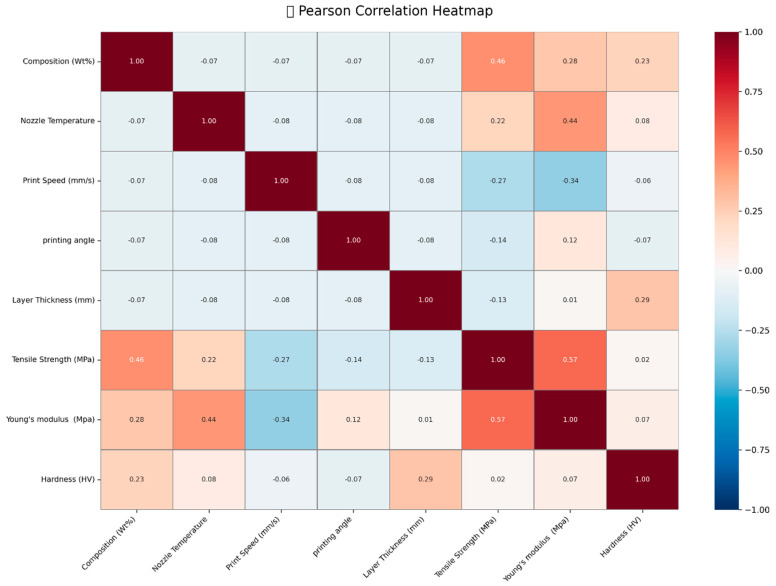
Correlation heat map of system variables related to responses.

The heatmap results reveal important relationships between processing parameters and mechanical properties, providing valuable insights for optimizing the performance of PLA-GN composites. Figure 18 presents a Pearson correlation heatmap, illustrating the relationships between various material properties and processing parameters. Composition (wt.%) shows a moderate positive correlation with tensile strength (0.46) and weak positive correlations with Young’s modulus (0.28) and hardness (0.23), indicating that changes in composition can slightly influence these properties. As GNP content increases, particularly at higher loadings (5 wt.%), the non-linear effects become more prominent due to agglomeration, which may further modify these correlations [36]. Nozzle temperature exhibits a weak positive correlation with tensile strength (0.22) and a moderate positive correlation with Young’s modulus (0.44), suggesting that it plays a role in enhancing material stiffness. Print speed demonstrates weak negative correlations with tensile strength (−0.27) and Young’s modulus (−0.34), implying that higher print speeds may reduce both strength and stiffness. Printing angle shows minimal correlations, with a weak negative correlation with tensile strength (−0.14) and a weak positive correlation with Young’s modulus (0.12). Layer thickness exhibits a weak positive correlation with hardness (0.29), suggesting that thicker layers may contribute to improved surface hardness. Tensile strength itself is moderately positively correlated with Young’s modulus (0.57) and weakly with hardness (0.29), indicating that stronger materials tend to be stiffer and slightly harder. Young’s modulus shows a weak positive correlation with hardness (0.07).

## 4. Conclusions

This study combines experimental, response surface methodology (RSM) and advanced machine learning (ML) models to optimize and predict the mechanical properties of 3D-printed PLA/GNP composites. The integrated approach provided valuable insights into the impact of printing parameters on mechanical performance, yielding the following key findings:

- Under optimal conditions (5% GNP content, nozzle temperature 210 °C, print speed 20 mm/s, and layer thickness 0.35 mm), the mechanical properties of PLA/GNP composites were significantly enhanced, showing a 67% improvement in tensile strength, a 205% increase in Young’s modulus, and a 40% improvement in hardness compared to pure PLA. These results were calculated relative to pure PLA as the baseline for comparison.
- The regression models produced an R^2^ of 99.35% and an Adjusted R^2^ of 97.3% securing highly predictive reliability. While high R^2^ values suggest a strong model fit, it is essential to acknowledge that overfitting can occur, particularly with small datasets. These models were validated using K-Fold Cross-Validation (K = 5), ensuring robust predictions and minimizing overfitting.
- Gaussian Process Regression and XGBoost demonstrated superior performance under the tested conditions, each exceeding R^2^ = 0.99 and achieving MAPE values below 4%. The Gaussian Process Regression model achieved an R^2^ of 0.9900 ± 0.0021, and XGBoost showed an R^2^ of 0.9716 ± 0.0103. These models demonstrated superior performance compared to linear regression, providing better predictive accuracy for the mechanical properties of PLA/GNP composites.
- SHAP feature importance analysis confirmed that GNP composition and layer thickness are the most influential contributors to the prediction of mechanical properties, with SHAP values reaching ±0.75, highlighting their critical roles in mechanical optimization.

This combined methodology of statistical design and interpretable machine learning enables robust process tailoring for high-performance, multifunctional 3D-printed composites, providing potential guidance for future material design and industrial applications. However, it is important to note that while the models provide valuable insights, they are based on experimental data and further validation with a broader range of data and application-specific scenarios is recommended [37,38,39].

## Figures and Tables

**Table 1:** Printing parameters, including composition.

S.No	Factor	Minimum	Maximum
1	Filler Composition (F_1_)	0.00 wt.%	5.00 wt.%
2	Temperature (F_2_)	190 °C	210 °C
3	Print Speed (F_3_)	20 mm/s	60 mm/s
4	Print Angle (F_4_)	0° (no rotation in the infill orientation)	90°
5	Layer Thickness (F_5_)	0.15 mm	0.35 mm

**Table 3:** Analysis of tensile strength.

Source	DF	Seq SS	Adj MS	F-Value	*p*-Value
Model	16	1440.93	90.06	47.77	0.0002
Composition (F_1_)	1	15.74	15.74	8.35	0.0342
Temperature (F_2_)	1	0.1397	0.1397	0.0741	0.7963
Print Speed (F_3_)	1	1.72	1.72	0.9097	0.3840
print angle (F_4_)	1	0.5178	0.5178	0.2746	0.6226
Layer Thickness (F_5_)	1	3.32	3.32	1.76	0.2416
F_1_*F_2_	1	83.92	83.92	44.51	0.0011
F_1_*F_3_	1	25.17	25.17	13.35	0.0147
F_1_*F_4_	1	13.14	13.14	6.97	0.0460
F_1_*F_5_	1	36.53	36.53	19.37	0.0070
F_2_*F_5_	1	21.59	21.59	11.45	0.0196
F_3_*F_4_	1	45.46	45.46	24.11	0.0044
F_3_*F_5_	1	145.36	145.36	77.10	0.0003
F_4_*F_5_	1	36.18	36.18	19.19	0.0072
F_1_^2^	1	150.40	150.40	79.77	0.0003
F_4_^2^	1	170.80	170.80	90.59	0.0002
F_5_^2^	1	26.30	26.30	13.95	0.0135
Residual	5	9.43	1.89		
Cor Total	21	1450.35			
R^2^ = 99.35%	R^2^(adj) = 97.27%				

Note: DF: Degrees of Freedom; Seq SS: Sequential Sum of Squares; Adj MS: Adjusted Mean Square; R^2^: Proportion of variance explained by the model; R^2^: Adjusted R^2^ accounting for the number of predictors; *: Interaction between factors.

**Table 4:** Analysis of Young’s modulus.

Source	DF	Seq SS (10^4^)	Adj MS (10^4^)	F-Value	*p*-Value
Model	15	879.7	58.64	40.41	<0.0001
Composition (F_1_)	1	5.171777	5.171777	3.56	0.108
Temperature (F_2_)	1	12.8	12.8	8.82	0.025
Print Speed (F_3_)	1	1.094886	1.094886	0.7545	0.4185
Print angle (F_4_)	1	1.006589	1.006589	0.6936	0.4368
Layer Thickness (F_5_)	1	91.47	91.47	63.03	0.0002
F_1_*F_2_	1	17.66	17.66	12.17	0.013
F_1_*F_3_	1	39.48	39.48	27.2	0.002
F_1_*F_4_	1	17.2	17.2	11.85	0.0138
F_1_*F_5_	1	37.16	37.16	25.61	0.0023
F_2_*F_3_	1	77.94	77.94	53.71	0.0003
F_2_*F_5_	1	27.75	27.75	19.12	0.0047
F_3_*F_4_	1	65.87	65.87	45.39	0.0005
F_4_*F_5_	1	43.36	43.36	29.88	0.0016
F_1_^2^	1	28.33	28.33	19.52	0.0045
F_5_^2^	1	83.45	83.45	57.5	0.0003
Residual	6	8.707293	1.451216		
Cor Total	21	888.4	58.64		
R^2^ = 99.02%	R^2^ (adj.) = 96.57%				

Note: DF: Degrees of Freedom; Seq SS: Sequential Sum of Squares; Adj MS: Adjusted Mean Square; R^2^: Proportion of variance explained by the model; R^2^: Adjusted R^2^ accounting for the number of predictors; *: Interaction between factors.

**Table 5:** Analysis of Hardness.

Source	DF	Seq. SS	Adj. MS	F-Value	*p*-Value
Model	12	2412.19	201.02	4.69	0.0134
Composition (F_1_)	1	338.68	338.68	7.89	0.0204
Temperature (F_2_)	1	0.4817	0.4817	0.0112	0.9179
Print Speed (F_3_)	1	140.52	140.52	3.28	0.1038
Print angle (F_4_)	1	9.13	9.13	0.2129	0.6555
Layer Thickness (F_5_)	1	503.53	503.53	11.74	0.0076
F_1_*F_5_	1	339.54	339.54	7.91	0.0203
F_2_*F_3_	1	187.56	187.56	4.37	0.0661
F_2_*F_4_	1	207.06	207.06	4.83	0.0556
F_2_^2^	1	610.25	610.25	14.22	0.0044
F_3_^2^	1	335.49	335.49	7.82	0.0208
F_4_^2^	1	494.63	494.63	11.53	0.0079
F_5_^2^	1	243.79	243.79	5.68	0.0410
Residual	9	386.12	42.90		
Cor Total	21	2798.31			
R^2^ = 86.20%	R^2^(adj) = 67.80%				

Note: DF: Degrees of Freedom; Seq SS: Sequential Sum of Squares; Adj MS: Adjusted Mean Square; R^2^: Proportion of variance explained by the model; R^2^: Adjusted R^2^ accounting for the number of predictors; *: Interaction between factors.

## Data Availability

The original contributions presented in this study are included in the article. Further inquiries can be directed to the corresponding author.
